# Exposure to intimate partner violence and lack of asthma control in adults: a cross-sectional study

**DOI:** 10.1590/1516-3180.2022.0336.R2.30032023

**Published:** 2023-06-09

**Authors:** Valmar Bião de Lima, Vanessa Serva Vazquez, Ana Clara Paixão Campos, Letícia Marques dos Santos, Álvaro Augusto Cruz

**Affiliations:** I BSc. Statistician and Researcher, Programa de Controle da Asma na Bahia (ProAR), Salvador (BA), Brazil; and Doctoral Student, Postgraduate Program in Medicine and Health, Universidade Federal da Bahia (UFBA), Salvador (BA), Brazil.; II PhD. Psychologist and Researcher, Programa de Controle da Asma na Bahia (ProAR),, Salvador (BA), Brazil.; III PhD. Doctoral Student, Centro de Integração de Dados e Conhecimentos para Saúde (CIDACS), Instituto Gonçalo Moniz, Fundação Oswaldo Cruz (FIOCRUZ), Salvador, Brazil.; IV PhD. Psychologist, Postgraduate Program in Collective Health, Universidade Federal da Bahia (UFBA), Salvador (BA), Brazil; and Associate Professor, Instituto de Humanidades Artes e Ciências (IHCA), Universidade Federal da Bahia (UFBA), Salvador (BA), Brazil.; V PhD. Physician, Universidade Federal da Bahia (UFBA), Salvador (BA), Brazil; CNPq Researcher 1 B, founder of the Program for the Control of Asthma in Bahia (ProAR) and Executive Director of the ProAR Foundation, Salvador (BA), Brazil.

**Keywords:** Depression, Mental health, Domestic violence, Intimate partner violence, Asthma, Asthma control, Conflict Tactics Scale, Physical aggression

## Abstract

**BACKGROUND:**

Asthma is a chronic airway disease that affects 339 million people worldwide. It is a heterogeneous disease with different risks, including in family environments, where intimate partner violence occurs.

**OBJECTIVE:**

This study aimed to investigate the possible association between psychosocial factors and asthma control in adults exposed to intimate partner violence.

**DESIGN AND SETTING:**

This cross-sectional study was conducted at a Brazilian public higher education institution in Salvador, Bahia, Brazil.

**METHODS:**

The study population consisted of adults clinically diagnosed with severe asthma and those with mild/moderate asthma identified at an asthma referral outpatient clinic. The sample comprised 492 participants who underwent clinical evaluation and completed questionnaires to assess asthma control, depression, stress, and resilience. The Conflict Tactics Scale, which measures tactics for managing marital conflicts, was used to estimate the level of intimate partner violence.

**RESULTS:**

Of the 492 participants, 76.2% were women and 91% self-referenced color black/brown, 37.8% reported low family income, 87.4% reported low education level, 71.7% reported high stress, 32.5% reported low resilience, 18.5% reported moderate or severe depression, 83.3% reported resolute negotiation, 49.4% reported major psychological aggression, 19.6% reported major physical aggression, 15.5% reported major injury, and 7.3% reported major sexual coercion. Regression analysis revealed that sex was an effect modifier.

**CONCLUSION:**

Women in situations of social vulnerability, with low income and poor education, with depression, severe asthma, and those who used aggression to resolve marital conflicts had a profile associated with a lack of asthma control.

## INTRODUCTION

Asthma is a chronic disease, which is typically characterized by increased responsiveness of the airways, with consequent obstruction of the airflow.Asthma is usually reversible spontaneously or with treatment. Its main symptoms are shortness of breath, wheezing, and tightness in the chest.^1^

According to the Global Initiative for Asthma (GINA), asthma is a heterogeneous and multifactorial disease with different pathological processes that affects 1–22% of the world’s population, regardless of age. Although the incidence of asthma among children has decreased in recent decades, it is estimated that 358 million individuals are affected worldwide. Furthermore, 495,000 deaths are caused by the disease annually.^2^ In Brazil, it is estimated that 12.4% of adults are diagnosed with asthma.^3^

Several factors contribute to the risk of developing asthma, including genetics, obesity, diet, female sex, occupational exposure, exposure to allergens, and high levels of family stress.^2^

Environmental and social stressors can affect different health conditions.^4,5^ Psychosocial stress alters the susceptibility to infectious and systemic diseases, which can increase airway inflammation in asthma, causing exacerbations.^6^

Intimate partner violence can be considered as a stressor related to asthma. There is evidence that stressful environments, wherein violent events are experienced, can significantly affect asthma control.^6,7^

There is evidence obtained through experimental and observational studies of the causal relationship between stress and poor asthma control; however, there is little evidence of causality between exposure to family, work, or community violence and asthma.^8^

The current World Health Organization definition of violence covers interpersonal violence, suicidal behavior, and armed conflict. It also encompasses a wide range of “acts,” going beyond physical assaults, including coercion and intimidation. The latter studies mainly investigated the relationship between aggressors and victims within the scope, while marital conflict was also investigated.^9^

The Conflict Tactics Scales (CTS2) assess the different ways couples lead conflict, considering how both manage it.Aggressive behavior as a conflict management tactic was assessed using one of the CTS2 subscales.

Marital violence has been studied in different contexts and centered on gender relations; however, it has been poorly studied along with chronic diseases, such as asthma. Therefore, it is necessary to investigate the dimensions of conjugal violence and spousal conflict management associated with asthma control. The CTS2 was used in this study. Therefore, disease control is essential to prevent crises and hospitalization.

## OBJECTIVE

Considering the relationships described in the literature between asthma and psychosocial factors, the aims of the present study were I) to describe CTS2 through the latent class analysis using the subscales of CTS2 in patients with asthma, II) assess the relationship between CTS2 and asthma control, and III) identify differences between sexes.

## METHODS

### Type of study

This is a cross-sectional study of data collected in the case-control study titled “Risk Factors, Biomarkers and Endophenotypes of Severe Asthma,” from Program for the Control of Asthma in Bahia (Programa de Controle da Asma, ProAR) – Universidade Federal da Bahia (UFBA). The participants were evaluated between 2013 and 2015 using ProAR. ProAR is the main reference center for specialized care in the treatment of severe asthma in Salvador.

In this case-control study, 1,448 participants were included. The severe asthma, no asthma, and mild/moderate asthma groups included 544, 454, and 450 participants, respectively. In this cross-sectional study, only participants with asthma who responded to the CTS2 and performed an assessment for asthma control were analyzed.

### Study population

The population considered included all participants in the case-control study. Participants with severe asthma were included from a ProAR cohort. Participants with mild/moderate asthma were recruited from the community. Posters were distributed in places of great circulation, including buses and places of primary care, where interviews were conducted in the waiting room or as indicated by the patients of the ProAR cohort.

The classification of patients with mild/moderate asthma was based on the concept of severity when the patient was evaluated in ProAR, including individuals with intermittent symptoms and without treatment or using low doses of controlling drugs upon evaluation by a specialist, following the criteria of the 2006 GINA,^10^ maintaining criteria similar to those of participants with severe asthma classified according to the 2002 GINA criteria.^11^

The diagnostic criteria were based on the presence of typical symptoms, improvement of symptoms with the use of bronchodilators or inhaled corticosteroids, and an increase in forced expiratory volume in 1 second by 12% and 200 ml after bronchodilator use.

### Sample, participants, and patients

A total of 492 volunteers with asthma were evaluated in multiple dimensions of their disease for possible risk factors and biomarkers, as described previously. In this study, a sample of 500 participants per group was estimated, and for a sample of 400 participants, we had at least 80% power.^12^

For the present study, the following inclusion and exclusion criteria were applied.

Inclusion Criteria: Patients aged above 18 years, residing in Salvador or in the metropolitan region, diagnosed with mild/moderate or severe asthma, who were in an intimate relationship in the last year, responded to the CTS2, and agreed to sign an informed consent form.

Exclusion Criteria: Pregnant women and patients with a history of serious illnesses, such as chronic obstructive pulmonary disease, or advanced neoplasia, that made it difficult to assess asthma control. Individuals who suffered a stroke (stroke), cardiac insufficiency, any other diseases that cause dyspnea; patients who suffered clinical complications that may interfere with the autonomy to answer questionnaires and that required a caregiver; and those who did not accept the signed informed consent form were also excluded.

The participants were aged ≥ 18 years, the diagnosis of asthma was confirmed by a specialist, and the individuals’ clinical history and current pulmonary function were considered. Therefore, as part of our protocol, spirometry tests were used to evaluate variable airflow obstruction, and chest radiography was performed to exclude other lung diseases.

Individuals who had previous smoking records ≥ 10 packs/year and those with other serious conditions that could confuse or interfere in the asthma diagnosis and asthma control were not included.

### Main measures, variables, and outcomes

Participants’ data were collected using an extensive standard form filled out by nurses and doctors. All relevant and general information, including sociodemographic variables, such as age, weight, height, income, education, and self-reported color, was registered.^13^

The following questionnaires validated in Brazil were applied to all individuals at baseline:1) Beck Scale for Depression;^14^ 2) Waldnig and Young’s scale of resilience;^15^ 3) Questionnaire to assess the level of psychological stress;^16^ 4) CTS2 was proposed and developed by Straus to identify different aspects of domestic violence with at least 1 year in a stable marital situation^17^ and validated for Portuguese.^18^

The CTS2 evaluated the variety of tactics used in response to the conflict with the partner but did not identify the causes of damage and health problems or the meaning of violent acts.^17,19^

It consisted of 78 items related to behaviors or experiences that configure different tactics to resolve marital conflict. Half of the items must be answered according to the tactics used by the interviewee; for the other half, the respondent must answer references in the tactics used by his partner/partner for conflict resolution.

The instrument consisted of five dimensions that characterize different conflict resolution tactics: negotiation (with two subscales: cognitive and emotional), psychological aggression (minor and major), physical aggression (minor and major), injury, herein referred to as damage and health problems (minor and major), and sexual coercion (minor and major). The cognitive subscale is the way in which partners use emotion for negotiation through conversation, whereas the emotional subscale is when emotion is used in an attempt to negotiate. The smallest subscale can be considered absent or mild, and the largest as severe.^17,20,21^ Notably, some of the conflict tactics characterized by the instrument corroborate the forms of violence from the perspective of current Brazilian law.^22^

### Statistical analysis

Descriptive statistics were calculated for all the variables. Bivariate analyses were performed using Chi-square and Fisher’s exact tests to identify associated and clinically relevant variables in relation to asthma control within the clinical context and violence between partners. The Mann–Whitney U test was used to verify the differences in body mass index (BMI) between the controlled and uncontrolled asthma groups. Latent class analysis (LCA) was used to describe the CTS2 of individuals on each CTS2 subscale to prevent participant overlap. Thus, the same participant was avoided from being allocated simultaneously to the subscales of the same dimension.

Questions 47, 48, 75, and 76, corresponding to the sexual coercion dimension of CTS2, were excluded from the LCA because there wasn’t at least one positive answer. The number of latent classes was determined using the Akaike information criterion, Bayesian information criterion, and entropy statistics for models with two, three, and four classes, respectively. However, the better results were those closer to 1.^23^ The best model was selected based on a combination of statistical criteria, parsimony, and interpretability of the latent classes. The assumption of local independence was assessed using a residual model (values < two were expected).

LCA has established itself as a useful statistical technique for grouping individuals into subtypes within a population when there is no prior knowledge about which individual belongs to which subpopulation.^23,24,25,26^ LCA is a method that uses the maximum likelihood estimation to form subgroups (latent classes), internally homogeneous and externally heterogeneous.^24^ The subgroups generated from LCA can be used to investigate the relationships with other characteristics, such as risk or protective factors.

Regression models were performed with dichotomized categorical predictor variables: physical aggression (minor and major), asthma severity (severe asthma and mild/moderate asthma), income (up to one salary or more than one salary), education (up to completing high school and at least not completing higher education), resilience (high and low), and depression (minimal/mild and severe/moderate). BMI was the only numerical predictor. Associations with other CTS2 dimensions were evaluated, but only the physical aggression dimension was relevant in the adjusted models.

SPSS (International Business Machines Corporation. Released 2012. IBM SPSS Statistics for Windows, Version 21.0. Armonk, New York, United States) was used to perform the descriptive analyses and hypothesis tests, and M-plus (Version 5. Computer Software. Los Angeles, California, United States: Muthén and Muthén) was used to obtain latent classes.

### Ethical aspects and financial source

We received funding from the National Council for Scientific and Technological Development (Conselho Nacional de Desenvolvimento Científico e Tecnológico, CNPq) support 471057/2014-2, the State of Bahia Foundation for Research Support (Fundação de Amparo à Pesquisa do Estado da Bahia, FAPESB), and GlaxoSmilthKline (an investigator-initiated grant). Additional support was provided by the Coordination for the Improvement of Higher Education Personnel, Brazil (Coordenação de Aperfeiçoamento de Pessoal de Nível Superior CAPES; Financing Code 001). It contemplated the preservation of the ethical and bioethical rights of the research subjects in accordance with the rules recommended in Resolution 466/2012 of the Council National Health Agency for Research in Human Beings. This project was approved by the Ethics Committee of the Maternidade Climério de Oliveira under OPINION/RESOLUTION No. 099/2009 on November 11, 2009, with additive opinion No. 032/2014.

## RESULTS

The participants’ main characteristics are listed in Table 1. Individuals with uncontrolled asthma were more likely to have severe asthma, a family income of up to one minimum wage, an education level of up to high school, a low level of resilience, severe or moderate depression, and a higher average BMI compared to those with controlled asthma.

**Table 1.** Association between sociodemographic characteristics and clinical variables with asthma control of the 492 participants included in the project “Risk factors, biomarkers and endophenotypes for severe asthma” between 2013 and 2015 who responded to conflict tactics scale (CTS2)

Variables	Controlled asthma n = 247 n (%)	Uncontrolled asthma n = 245 n (%)	Total n = 492 n (%)	P-value^&^
**Severity of asthma**				0.000
Moderate asthma	137 (55.5)	84 (34.3)	221 (44.9)	
Severe asthma	110 (44.5)	161 (65.7)	271 (55.1)	
**Older people**				0.321
No	220 (89.1)	211 (86.1)	431 (87.6)	
Yes	27 (10.9)	34 (13.9)	61 (12.4)	
**Gender**				0.490
Female	185 (74.9)	190 (77.6)	375 (76.2)	
Male	62 (25.1)	55 (22.4)	117 (23.8)	
**Family income ≤ minimum wage n = 460^#^**				0.002
No	157 (69.5)	129 (55.1)	286 (62.2)	
Yes	69 (30.5)	105 (44.9)	174 (37.8)	
**Self-referenced color black/brown**				0.409
No	19 (7.7)	24 (9.8)	43 (8.7)	
Yes	228 (92.3)	221 (90.2)	449 (91.3)	
**Education level ≤ high school**				0.016
No	40 (16.2)	22 (9.0)	62 (12.6)	
Yes	207 (83.8)	223 (91.0)	430 (87.4)	
**Perception level of stress**				0.213
Low	76 (30.8)	63 (25.7)	139 (28.3)	
High	171 (69.2)	182 (74.3)	353 (71.7)	
**Resilience level**				0.002
High	183 (74.1)	149 (60.8)	332 (67.5)	
Low	64 (25.9)	96 (39.2)	160 (32.5)	
**Moderate or severe depression**				0.000
No	219 (88.7)	182 (74.3)	401 (81.5)	
Yes	28 (11.3)	63 (25.7)	91 (18.5)	
Body mass index (μ±σ)*	28.0 ± 5.1	29.4 ± 5.7	28.7 ± 5.4	0.004**

^&^Chi-square test; **Mann–Whitney test; *Mean and standard deviation; ^#^Family income.

Regarding the LCA, two models were chosen because models with more than two classes hindered the interpretation and concept of each subscale and the characterization of the patients’ profiles according to the subscales of the CTS2 (https://documentcloud.adobe.com/link/review?uri=urn http://www.:aaid:scds:US:282ef302-057a-4291-992a-258baa9fcee0). The subscales for the dimensions of psychological aggression, physical aggression, injury, and sexual coercion were determined as described by Moraes, Paiva, and Figueiredo,^16,23^ except for the negotiation dimension, where the cognitive negotiation subscale was replaced by resolutive negotiation as both the participants and their partners had a high probability of a positive answer to the cognitive negotiation and emotional negotiation questions.

Analysis of the CTS2 subscales revealed the conflicting tactics adopted for intimate partner conflict management. As shown in Table 2, 82.4% of participants reported resolutive negotiations, 46.4% reported major psychological aggression, 15.2% reported minor physical aggression, 10.9% reported major injury, and 11.9% reported sexual coercion. According to them, 65.1% of their partners used resolutive negotiations, 39.6% used psychological aggression, 15.9% used physical aggression, 6.2% used injury, and 7.3% used sexual coercion (https://documentcloud.adobe.com/link/review?uri=urn:aaid:scds:US:282ef302-057a-4291-992a-258baa9fcee0).

**Table 2.** Association between the domains of the conflict tactics scale (CTS2) and the asthma control of 375 female, 117 male, and total 492 participants included in the project “Risk factors: biomarkers and endophenotypes of severe asthma” between 2013 and 2015

Domains	Controlled asthma female n = 185	Uncontrolled asthma female n = 190	P valor^&^	Controlled asthma male n = 62	Uncontrolled asthma male n = 55	P-valor^&^	Controlled asthma n = 247	Uncontrolled asthma n = 2 45	P valor^&^
Participant									
**Negotiation**			0.782			0.914			0.776
Resolutive	152 (82.2)	154 (81.1)		56 (90.3)	50 (90.9)		208 (84.2)	204 (83.3)	
Emotional	33 (17.8)	36 (18.9)		6 (9.7)	5 (9.1)		39 (15.8)	41 (16.7)	
**Psychological aggression**			0.384			0.522			0.242
Minor	94 (50.8)	88 (46.3)		44 (71.0)	36 (65.5)		138 (55.9)	124 (50.6)	
Severe	91 (49.2)	102 (53.7)		18 (29.0)	19 (34.5)		109 (44.1)	121 (49.4)	
**Physical aggression**			0.009			0.298^#^			0.005
Minor	163 (88.1)	148 (77.9)		58 (93.5)	49 (89.1)		221 (89.5)	197 (80.4)	
Severe	22 (11.9)	42 (22.1)		4 (6.5)	6 (10.9)		26 (10.5)	48 (19.6)	
**Sexual coercion**			0.502			0.189^#^			0.980
Minor	170 (91.9)	178 (93.7)		59 (95.2)	49 (89.1)		229 (92.7)	227 (92.7)	
Severe	15 (8.1)	12 (6.3)		3 (4.8)	6 (10.9)		18 (7.3)	18 (7.3)	
**Injury**			0.051			0.573^#^			0.053
Minor	165 (89.2)	156 (82.1)		58 (93.5)	51 (92.7)		223 (90.3)	207 (84.5)	
Severe	20 (10.8)	34 (17.9)		4 (6.5)	4 (7.3)		24 (9.7)	38 (15.5)	
**Partner**									
**Negotiation**			0.060			0.716			0.058
Resolutive	130 (70.3)	116 (61.1)		48 (77.4)	41 (74.5)		178 (72.1)	157 (64.1)	
Emotional	55 (29.7)	74 (38.9)		14 (22.6)	14 (25.5)		69 (27.9)	88 (35.9)	
**Psychological aggression**			0.124			0.533			0.239
Minor	111 (60.0)	99 (52.1)		43 (69.4)	41 (74.5)		154 (62.3)	140 (57.1)	
Severe	74 (40.0)	91 (47.9)		19 (30.6)	14 (25.5)		93 (37.7)	105 (42.9)	
**Physical aggression**			0.167			0.811			0.164
Minor	158 (85.4)	152 (80.0)		55 (88.7)	48 (87.3)		213 (86.2)	200 (81.6)	
Severe	27 (14.6)	38 (20.0)		7 (11.3)	7 (12.7)		34 (13.8)	45 (18.4)	
**Sexual coercion**			0.423			0.046^#^			0.809
Minor	175 (94.6)	183 (96.3)		62 (100.0)	51 (92.7)		237 (96.0)	234 (95.5)	
Severe	10 (5.4)	7 (3.7)		–	4 (7.3)		10 (4.0)	11 (4.5)	
**Injury**			0.588			0.721^#^			0.577
Minor	180 (97.3)	183 (96.3)		61 (98.4)	54 (98.2)		241 (97.6)	237 (96.7)	
Severe	5 (2.7)	7 (3.7)		1 (1.6)	1 (1.8)		6 (2.4)	8 (3.3)	

^&^Chi-square test; ^#^Fisher’s exact test.

Although there was no association between the domain of negotiation and asthma control, high percentages of its use were observed regardless of sex; that is, the use of other conflict tactics jointly involves resolutive negotiation, in which the couple tries to resolve their conflicts through conversation.

According to the stratified analysis by sex, a vulnerability profile was identified for women in relation to the lack of asthma control (Table 3).

**Table 3.** Logistic regression analysis for association between psychosocial factors, health status, and asthma control stratified by sex

Covariates	Crude OR (CI 95%)	Adjusted OR (CI 95%)
Female		
Physical aggression	2.103 (1.199–3.687)	1.967 (1.038–3.728)
Severe asthma	2.575 (1.697–3.908)	2.647 (1.691–4.145)
Family income ≤ minimum wage	1.793 (1.169–2.752)	1.770 (1.126–2.780)
Education level ≤ high school	2.189 (1.153–4.156)	1.264 (0.613–2.607)
Body mass index	–	1.032 (0.992–1.075)
Resilience low	1.764 (1.150–2.707)	1.436 (0.870–2.371)
Moderate or severe depression	2.882 (1.711–4.856)	2.354 (1.323–4.190)
Male		
Physical assault	1.776 (0.474–6.654)	2.230 (0.533–9.334)
Severe asthma	1.964 (0.920–4.196)	2.265 (1.003–5.114)
Family income ≤ minimum wage	2.117 (0.834–5.376)	2.204 (0.804–6.044)
Education level ≤ high school	1.387 (0.460–4.181)	1.384 (0.388–4.940)
Body mass index	–	1.080 (0.979–1.191)
Resilience low	2.133 (0.874–5.208)	2.207(0.874–5.573)
Moderate or severe depression	1.542 (0.330–7.218)	1.120 (0.221–5.683)

OR = odds ratio; CI = confidence interval. Both models were fitted using physical assault, asthma severity, family income, educational level, body mass index, resilience, and depression.

The use of physical assault tactics, severe asthma, low education, low income, low resilience, and moderate or severe depression determined asthma control. However, this was not observed in men, where only the severity of asthma increased the risk of having uncontrolled asthma. Several regression models were used to investigate the conflict tactic management used by partners, but no associations were found.

## DISCUSSION

According to the results, 20% of the participants reported the use of physical aggression, injury, and sexual coercion to deal with conflicts within marital relationships. Although these tactics have been reported by a minority of individuals, mainly women, they still require special attention in health services because the real dimensions of women’s psychological suffering and how a violent profile impacts chronic diseases, such as asthma, are unknown.

Although the results of the present study showed that women used aggression to resolve conflicts with their intimate partners, it is known that the greatest burden of violence falls on them. Women have been the main victims of men over time^27^ and also those who have suffered great damage to their health.^4^

Despite the literature showing associations between risk factors for asthma and its control,^4–6^ there is little evidence on the relationship between spousal violence and asthma. However, it was identified that women who suffered violence, whether in the past or recently, were at a high risk of developing asthma. In line with the present study, a stratified analysis by sex and age showed that women in the age groups below 44 years needed to use physical aggression as a conflict resolution tactic and that they were also at a high risk of uncontrolled asthma.^7^

Our results reinforce the psychosocial vulnerability of this population and demonstrate its impact on marital relationships. It is noteworthy that the ability to be resilient is related to sociodemographic characteristics, such as high schooling and high income,^28^ and study participants had the opposite characteristics. In addition, we believe that depression reveals psychological suffering in this population. These findings are also addressed by other authors who demonstrated the relationship between intimate partner violence and psychological stress with a lack of asthma control and psychosocial aspects.^27,29^

Another important outcome relates to negotiations as a conflict resolution tactic. Although there was no statistical relevance, the high percentages in both the controlled and uncontrolled groups indicated that both partners used this tactic to resolve marital conflict. The most frequent form was resolutive negotiation, but it was probably ineffective in dealing with conflicts if we consider the high percentages of aggression reported by women.

The use of aggression as a form of conflict may be associated with other unstudied factors, such as irritability, which may be related to depression and lack of control over asthma. The association among uncontrolled asthma, parental depression, and family chaos, including commotion, disorganization, and routine at home, should be considered.^30^

Our findings point to the need for studies, whether quantitative or qualitative, to better understand how each of these psychosocial characteristics is related to asthma control, as well as the etiopathogenesis of the disease. Psychosocial factors play an important role in asthma, either as precipitating elements of exacerbation or disease progression, showing that a poor perception of physical control is associated with a poor quality of life in asthmatics.^31^

In contrast, asthma itself has an impact on psychosomatic responses, as it involves biological and psychological factors directly linked to interpersonal relationships and social bonds in many ways. Asthma impacts the quality of life. The disease is related to difficult experiences permeated by suffering for the patient.^31^ This is supported by our results, which revealed that depression (severe or moderate), low resilience, and physical aggression were associated with a lack of asthma control.

This study has some limitations. The first relates to reverse causality. It is impossible to state the temporality of the association between depression and the lack of asthma control. However, depression may also be related to recurrent breathing difficulties. We also considered a vicious cycle in which there was bidirectional causality between asthma and depression. Second, the sample of volunteers interfered with the external validity. Therefore, the findings can only be extrapolated to parsimony.

Despite these limitations, it is important to highlight the use of LCA, which is a poorly used technique in Brazil. However, it is important to define variables that are not directly observable. The use of latent classes within the health context was also important in identifying those that would be correlated with other variables in the adjustment of the regression models.

In addition, although CTS2 is a validated questionnaire with extensive bibliographic support, the results might be influenced by interview bias, as people with depression already have a negative view of the facts, which may overestimate the use of physical aggressive tactics and underestimate such occurrences by not reporting aggression.

## CONCLUSIONS

In the present study, women in situations of social vulnerability, with low income and poor education, with depression, severe asthma, and those who used aggression as a means of resolving marital conflicts had a profile associated with a lack of asthma control. Although CTS2 does not clarify the origin of violence, it is understood that its use of physical aggression tactics is in response to a conflict that occurred previously. Further studies are needed to better assess the relationship between domestic violence, mental health, and asthma and to explore its causality. Multiprofessional health teams, especially referral centers for severe asthma, should consider the importance of marital relationships and depression in asthma control and seek interventions that contribute to the development of effective and nonviolent conflict resolutions.
